# Cetuximab promotes RSL3-induced ferroptosis by suppressing the Nrf2/HO-1 signalling pathway in KRAS mutant colorectal cancer

**DOI:** 10.1038/s41419-021-04367-3

**Published:** 2021-11-13

**Authors:** Jiawen Yang, Jiajie Mo, Juji Dai, Chenqiao Ye, Wei Cen, Xuzhi Zheng, Lei Jiang, Lechi Ye

**Affiliations:** 1 Department of Colorectal and Anal Surgery, The First Affiliated Hospital of Wenzhou Medical University, 325000 Wenzhou, People’s Republic of China; 2 Central Laboratory, The First Affiliated Hospital of Wenzhou Medical University, 325000 Wenzhou, People’s Republic of China

**Keywords:** Targeted therapies, Cell death

## Abstract

Cetuximab is approved for the treatment of metastatic colorectal cancer (mCRC) with RAS wild-type. Nevertheless, the prognosis remains poor and the effectiveness of cetuximab is limited in KRAS mutant mCRC. Recently, emerging evidence has shown that ferroptosis, a newly discovered form of nonapoptotic cell death, is closely related to KRAS mutant cells. Here, we further investigated whether cetuximab-mediated regulation of p38/Nrf2/HO-1 promotes RSL3-induced ferroptosis and plays a pivotal role in overcoming drug resistance in KRAS mutant colorectal cancer (CRC). In our research, we used two KRAS mutant CRC cell lines, HCT116 and DLD-1, as models of intrinsic resistance to cetuximab. The viability of cells treated with the combination of RSL3 and cetuximab was assessed by the CCK-8 and colony formation assays. The effective of cetuximab to promote RSL3-induced ferroptosis was investigated by evaluating lipid reactive oxygen species accumulation and the expression of the malondialdehyde and the intracellular iron assay. Cetuximab therapy contributed to regulating the p38/Nrf2/HO-1 axis, as determined by western blotting and transfection with small interfering RNAs. Cetuximab promoted RSL3-induced ferroptosis by inhibiting the Nrf2/HO-1 in KRAS mutant CRC cells, and this was further demonstrated in a xenograft nude mouse model. Our work reveals that cetuximab enhances the cytotoxic effect of RSL3 on KRAS mutant CRC cells and that cetuximab enhances RSL3-induced ferroptosis by inhibiting the Nrf2/HO-1 axis through the activation of p38 MAPK.

## Introduction

Colorectal cancer (CRC) is the third most deadly cancer in the world [1, 2]. Despite the development of diagnostic and therapeutic strategies, CRC remains one of the most severe human cancers due to resistance to treatment.

In recent years, great progress has been made in the combination of cetuximab and chemotherapy in the treatment of metastatic colorectal cancer (mCRC) [3]. However, KRAS mutations are seen in 36–46% of CRC cases, and they do not benefit from cetuximab treatment [4, 5]. Treatment with cetuximab is ineffective in CRC patients with KRAS mutations [6, 7]. Therefore, we considered how to develop the therapeutic efficacy of cetuximab in KRAS mutant CRC in our study.

Ferroptosis is a newly discovered form of nonapoptotic cell death that is triggered via iron-dependent lipid peroxidation [8]. The mechanisms of ferroptosis are distinct from those of typical cell death processes, such as necrosis, autophagy, and apoptosis [9]. RAS-selective lethal 3 (RSL3), a small molecule, can kill RAS mutant cancer cells [10] and activate iron-dependent, nonapoptotic cell death of RAS mutant cancer cells [11]. RSL3 is a potent ferroptosis-triggering agent that directly inhibits GPX4, which can promote ferroptosis, including CRC cells [12]. Thus, there might be special links between ferroptosis and KRAS mutant CRC.

Nuclear factor erythroid 2-related factor 2 (Nrf2), a dominant regulator of antioxidant transcription factors, prevents lipid peroxidation [13] and ferroptosis by increasing the transcription of multiple cytoprotective enzymes, such as haem oxygenase-1 (HO-1) [14]. Ferroptosis can be alleviated potently via Nrf2/HO-1 axis activation through the abolishment of lipid oxidation [15, 16]. Therefore, suppressing Nrf2/HO-1 signalling could be a potential target in promoting ferroptosis. p38 mitogen-activated protein kinase (p38 MAPK) can be activated by cetuximab [17], and p38 MAPK has been confirmed to be involved in the regulation of Nrf2/HO-1 [18, 19]. Accordingly, we speculated that activation of p38 MAPK plays a major role in suppressing Nrf2/HO-1 signalling.

Here, our study showed that combined treatment with RSL3 and cetuximab increases the death of HCT116 and DLD-1 KRAS mutant CRC cell lines, leading to the accumulation of lipid peroxide products and decreased cell viability. Significantly, we for the first time demonstrated that cetuximab can promote RSL3-induced ferroptosis by markedly suppressing Nrf2/HO-1 signalling *via* p38 MAPK activation in KRAS mutant CRC cell lines.

## Results

### Cetuximab enhances the cytotoxicity effect of RSL3 treatment on KRAS mutant CRC cells

**Fig. 1:**
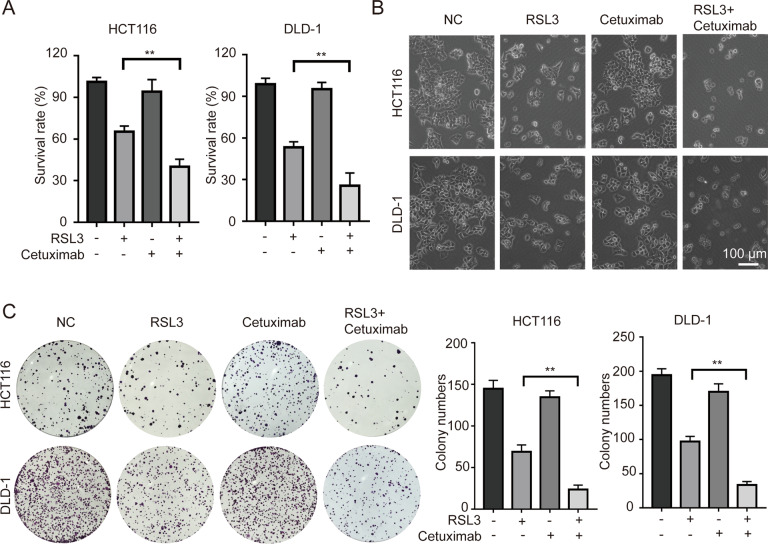
Cetuximab enhances the cytotoxic effect of RSL3 on KRAS mutant CRC cells. **A** HCT116 and DLD-1 cells were treated with RSL3 (1 μM), cetuximab (100 μg/ml) or their combination for 24 h. The inhibitory effects were determined by the CCK-8 assay. **B** Cell morphology changes were observed by inverted light microscopy. Scale bar, 100 μm. **C** The colony formation with the indicated treatment was performed (left panel), and a histogram of colony numbers is shown (right panel). **P < 0.01.

Four KRAS mutant CRC cell lines (HCT116, DLD-1, LOVO and SW480) were treated in indicated concentration of RSL3 alone or in combination with cetuximab (100 μg/ml) for 24 h. (Fig. S1). We found that combination treatment with RSL3 and cetuximab enhanced the death of HCT116 and DLD-1 cells compared with RSL3 alone. In subsequent experiments, HCT116 and DLD-1 cells were chosen and treated with RSL3 (1 μM), cetuximab (100 μg/ml) for 24 h. We found that treatment with cetuximab enhanced the cytotoxic effect of RSL3 in HCT116 and DLD-1 cells (Fig. 1A, B).

We next investigated whether cetuximab enhances antiproliferative effects on RSL3-treated HCT116 and DLD-1 cells, and the colony formation assay was performed. Compared with RSL3 or cetuximab alone, combination treatment with RSL3 and cetuximab significantly suppressed proliferation (Fig. 1C). All these results suggest that cetuximab enhances the cytotoxic effect of RSL3 on KRAS mutant CRC cell lines by significantly inhibiting cell viability and inhibiting cell proliferation.

### Cetuximab enhances RSL3-induced ferroptosis in KRAS mutant CRC cells

**Fig. 2:**
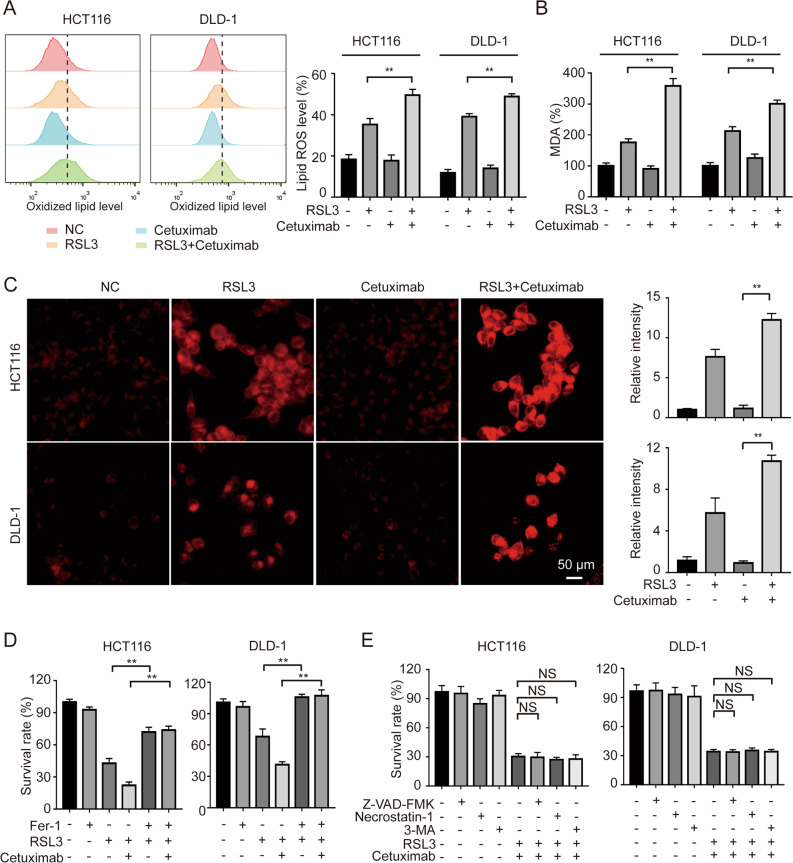
Cetuximab treatment enhances RSL3-induced ferroptosis in KRAS mutant CRC cells. **A** HCT116 and DLD-1 cells were treated with RSL3 (1 μM), cetuximab (100 μg/ml) or their combination for 24 h. The cellular lipid ROS level was analyzed by a flow cytometer. **B** HCT116 and DLD-1 cells were treated with RSL3 (1 μM), cetuximab (100 μg/ml) or their combination for 24 h. Intracellular MDA levels were measured by an MDA Assay Kit. **C** HCT116 and DLD-1 cells were treated with RSL3 (1 μM), cetuximab (100 μg/ml) or their combination for 24 h. Chelatable iron levels were determined using FerroOrange. Scale bar, 50 μm. **D** HCT116 and DLD-1 cells were treated with cetuximab (100 μg/ml) or RSL3 (1 μM) in the absence or presence of Fer-1 (1 μM) for 24 h, and cell viability was evaluated by the CCK-8 assay. **E** HCT116 and DLD-1 cells were treated with cetuximab (100 μg/ml) or RSL3 (1 μM) in combination with Z-VAD-FMK (10 μM), necrostatin-1 (10 μM) or 3-MA (60 μM) for 24 h, and cell viability was assessed by the CCK-8 assay. **P < 0.01; *P < 0.05; NS: no significance.

RSL3 is a potent ferroptosis activator [20]. To investigate whether ferroptosis was further promoted by treatment with RSL3 and cetuximab, two indicators of ferroptosis, i.e., lipid reactive oxygen species (ROS) accumulation and malondialdehyde (MDA) levels, were assessed in KRAS mutant HCT116 and DLD-1 cells. Then, C11‐BODIPY^581/591^, a lipid peroxidation probe, was applied to measure the level of intracellular lipid ROS. Consistent with our expectation, following co-treatment with RSL3 and cetuximab, lipid ROS accumulation was remarkably increased (Fig. 2A). Moreover, MDA levels were remarkably increased after co-treatment with RSL3 and cetuximab in HCT116 and DLD-1 cells (Fig. 2B). Intriguingly, treatment with cetuximab did not affect lipid ROS accumulation or MDA levels in either HCT116 or DLD-1 cell lines but enhanced the RSL3-induced increases in lipid ROS levels and MDA levels. These results indicate that ferroptosis might be promoted after co-treatment with RSL3 and cetuximab in KRAS mutant CRC cell lines. Ferroptosis is characterized by iron-dependent accumulation [9]. Thus, intracellular iron levels were determined in HCT116 and DLD-1 cells by using a Fe^2+^ iron probe known as FerroOrange. The cells co-treated with RSL3 and cetuximab emitted much stronger orange fluorescence compared with treated with RSL3 alone (Fig. 2C).

Moreover, treatment with RSL3 and cetuximab increased the inhibition of HCT116 and DLD-1 cell growth, and this effect was reversed by the ferroptosis rescue agent Fer-1 (Fig. 2D) but not by the rescue agent of apoptosis (Z-VAD-FMK), necroptosis (necrostatin-1), or autophagy (3-MA) (Fig. 2E). Collectively, these findings strongly suggest that cetuximab enhances RSL3-induced ferroptosis in KRAS mutant CRC cell lines.

### Combination treatment with RSL3 and cetuximab inhibits Nrf2/HO-1 axis in KRAS mutant CRC cells

**Fig. 3:**
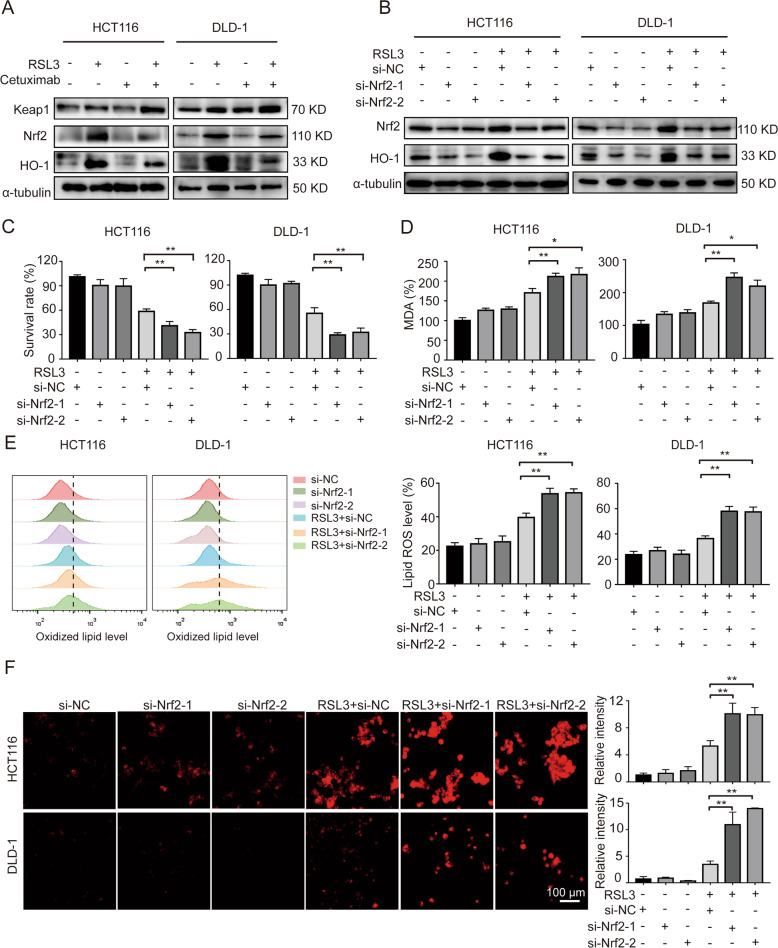
Combination treatment with RSL3 and cetuximab inhibits Nrf2/HO-1 axis in KRAS mutant CRC cells. **A** The protein levels of Keap1, Nrf2 and HO-1 in HCT116 and DLD-1 cells were measured by western blotting after treatment with RSL3 (1 μM), cetuximab (100 μg/ml) or their combination for 24 h. **B** Knockdown of Nrf2 by siRNA reduced the expression of Nrf2 and the protein levels of Nrf2 and HO-1 after treatment with RSL3 (1 μM) for 24 h. **C** siRNA-mediated knockdown of Nrf2 increased the sensitivity of HCT116 and DLD-1 cells to RSL3. **D**–**F** The levels of MDA, lipid ROS and intracellular iron were measured in Nrf2-silenced HCT116 and DLD-1 cells pretreated with or without RSL3 (1 μM). Scale bar, 100 μm. **P < 0.01; *P < 0.05.

Kelch-like ECH-associated protein-1 (Keap1) is a master regulator in cellular responding to oxidative stress. Following oxidative stress, Keap1 is dissociated from Nrf2, leading to Nrf2 accumulation, nuclear translocation, and activate the transcription of target gene including HO-1 [21]. Our study found that following treatment with RSL3 and cetuximab increasing Keap1 expression, and inhibiting Nrf2/HO-1 signalling, as shown by western blot analysis (Fig. 3A).

To evaluate the effects of the expression of Nrf2 on KRAS mutant CRC cells treated with cetuximab and RSL3, we designed two small interfering RNAs (siRNAs) targeting Nrf2. In HCT116 and DLD-1 cells, siRNA transfection for 48 h period reduced the expression of Nrf2 and its downstream target HO-1. Compared with the RSL3 group, the Nrf2 and HO-1 level in the RSL3-treated transfected groups was reduced (Fig. 3B). Transfection of an siRNA targeting Nrf2 increased the sensitivity of HCT116 and DLD-1 cells to RSL3 (Fig. 3C).

We sought to further confirm whether knockdown of Nrf2 can cause lipid oxidation, and we measured the levels of MDA and intracellular ROS in Nrf2-siRNA-transfected HCT116 and DLD-1 cells. As expected, knockdown of Nrf2 enhanced RSL3-induced MDA levels (Fig. 3D). Then, we used flow cytometry to analyze the intracellular lipid ROS level, and the results demonstrated that knockdown of Nrf2 promoted RSL3-induced oxidation of lipids in HCT116 and DLD-1 cells (Fig. 3E). Additionally, the results showed that knockdown of Nrf2 enhanced RSL3-induced intracellular iron accumulation (Fig. 3F). These results indicate that combination treatment with RSL3 and cetuximab inhibits Nrf2/HO-1 signalling in KRAS mutant CRC cells.

### Cetuximab activates p38 MAPK and regulates the Nrf2/HO-1 axis

**Fig. 4:**
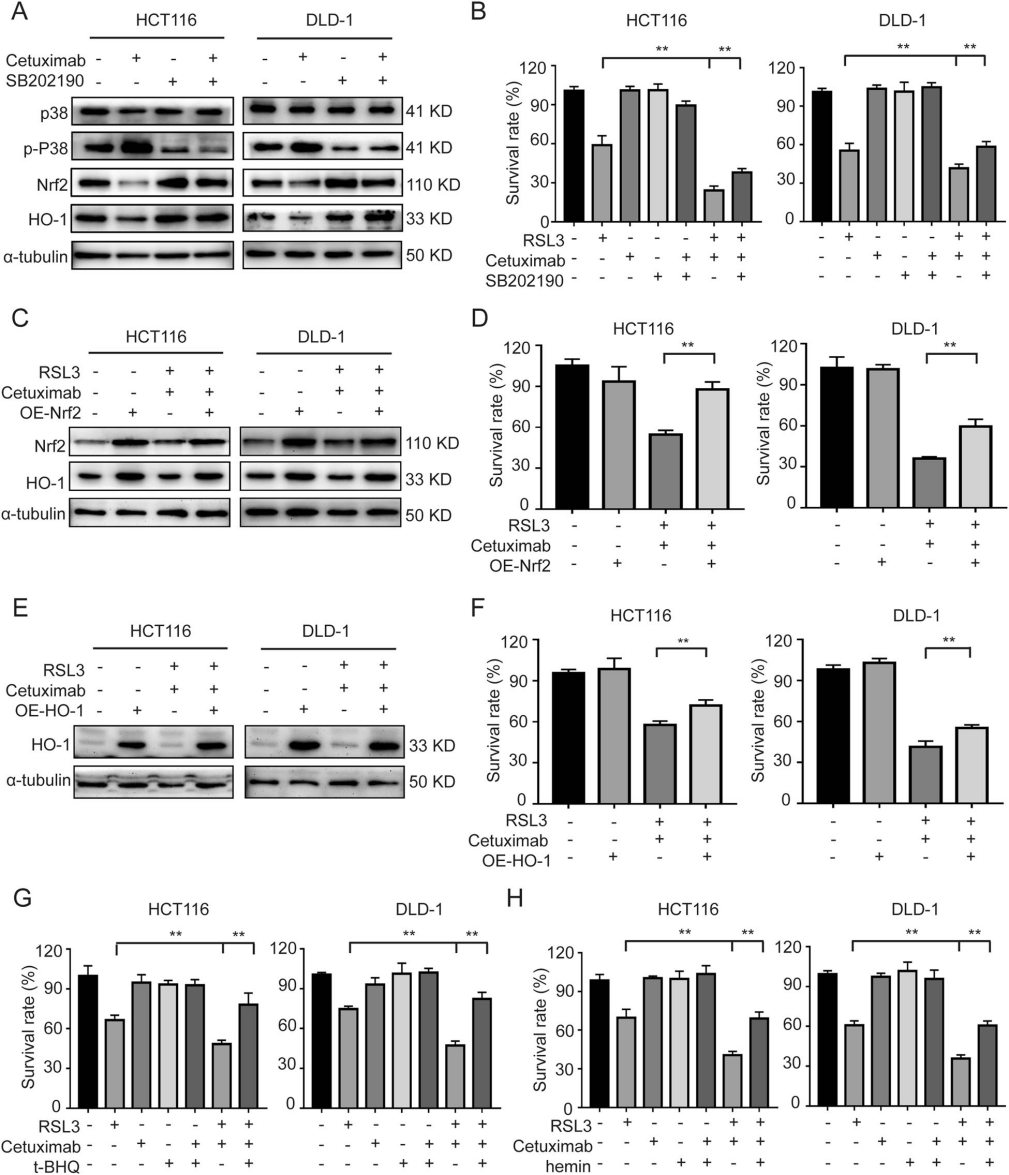
Cetuximab activates p38 MAPK and regulates the Nrf2/HO-1 axis. **A** Western blot analysis of p-p38, total p38, Nrf2 and HO-1 expression levels in HCT116 and DLD-1 cells incubated with cetuximab (100 μg/ml), SB202190 (1 μM) or cetuximab in combination with SB202190 for 24 h. **B** HCT116 and DLD-1 cells were treated with cetuximab (100 μg/ml) or RSL3 (1 μM) in the absence or presence of SB202190 (1 μM) for 24 h, and cell viability was assessed by the CCK-8 assay. **C** The protein levels of Nrf2 and HO-1 in HCT116 and DLD-1 cells or Nrf2 overexpressed HCT116 and DLD-1 cells treated with RSL3 (1 μM) combination with cetuximab (100 μg/ml) for 24 h. **D** HCT116 and DLD-1 cells with overexpression Nrf2 were treated with or without RSL3 (1 μM) combination with cetuximab (100 μg/ml) for 24 h. Cell viability was assessed by CCK-8 assays. **E** The protein level of HO-1 in HCT116 and DLD-1 cells or HO-1 overexpressed HCT116 and DLD-1 cells treated with RSL3 (1 μM) combination with cetuximab (100 μg/ml) for 24 h. **F** HCT116 and DLD-1 cells with overexpression HO-1 were treated with or without RSL3 (1 μM) combination with cetuximab (100 μg/ml) for 24 h. Cell viability was assessed by CCK-8 assays. **G**–**H** HCT116 and DLD-1 cells were treated with cetuximab (100 μg/ml) or RSL3 (1 μM) with or without t-BHQ (20 μM) or hemin (20 μM) for 24 h, and cell viability was assessed by the CCK-8 assay. **P < 0.01.

p38 MAPK has been reported to regulate antioxidant enzymes expression, including Nrf2 and HO-1 [22]. To investigate the role of p38 MAPK in the expression of Nrf2/HO-1, we evaluated the effect of cetuximab and SB202190 (specific inhibitors of p38) on p38 activity in HCT116 and DLD-1 cells. The protein level of p38 in cetuximab- or SB202190-treated cells was not different from that in control. The levels of p-p38 were significantly enhanced by cetuximab treatment, but this effect was abolished when co-treatment with cetuximab and SB202190 (Fig. 4A). We then investigated whether inhibition of p38 expression can affect cetuximab-induced inhibition of Nrf2 and HO-1 expression. Western blot analysis demonstrated that treatment with cetuximab decreased the expression of Nrf2 and HO-1, and SB202190 reduced cetuximab-mediated inhibition of Nrf2 and HO-1 (Fig. 4A). Moreover, the death of HCT116 and DLD-1 cells induced by treatment with RSL3 and cetuximab was partly rescued by SB202190 (Fig. 4B). To further verify if Nrf2/HO-1 pathway is involved in which cetuximab sensitizes cells to RSL3, HCT116 and DLD-1 cells were transient transfected with Nrf2 or HO-1 expressing vectors. As shown in Fig. 4C, D, overexpression of Nrf2 restored the combination treatment with cetuximab and RSL3-induced inhibition of HO-1 expression and cell growth in HCT116 and DLD-1 cells. Also, overexpression of HO-1 reversed the growth inhibition induced by cetuximab and RSL3 combination treatment in CRC cells (Fig. 4E, F). In addition, activation of Nrf2 by t-BHQ treatment or activation of HO-1 by hemin treatment partly reversed the growth inhibition of HCT116 and DLD-1 cells induced by RSL3 and cetuximab treatment (Fig. 4G, H). In addition, knockdown of Keap1 significantly restored theNrf2 and HO-1, and reduced the cytotoxicity induced by co-treatment cetuximab and RSL3 in HCT116 and DLD-1 cells (Fig. S2). These results demonstrate that p38 MAPK participates in the regulation of Nrf2 and HO-1 expression and that cetuximab can inhibit the Nrf2/HO-1 pathway *via* p38 MAPK activation.

### Cetuximab enhances RSL3-induced ferroptosis by suppressing the Nrf2/HO-1 axis in KRAS mutant CRC cells in a xenograft nude mouse model

**Fig. 5:**
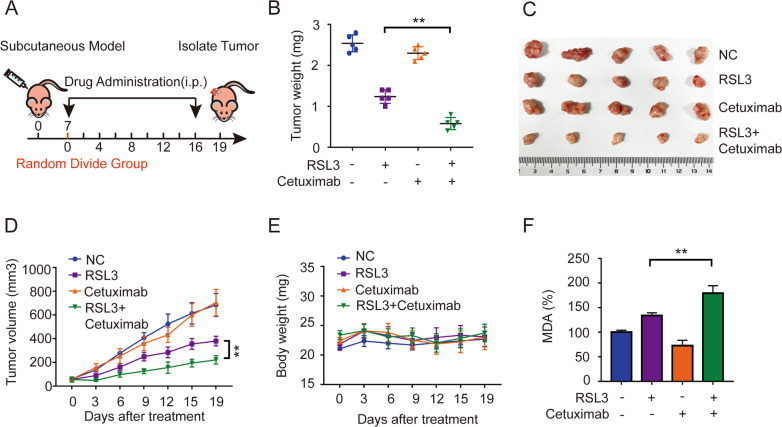
Cetuximab enhances the inhibitory effects of RSL3 and RSL3-induced ferroptosis in vivo. **A** Schematic of the anticancer effect of the combination of RSL3 (5 mg/kg) and cetuximab (13 mg/kg) in a DLD-1 cell subcutaneous tumour model. **B** The tumour weights of excised tumours on day 19. **C** Excised tumours on day 19. **D** The tumour volume of each group was calculated every 3 days. **E** The body weight of each group was calculated every 3 days. **F** The level of MDA in excised tumours on day 19 was assayed. **P < 0.01.

To further explored whether cetuximab promotes RSL3-induced ferroptosis in vivo, a DLD-1 xenograft nude mouse model was established. A schematic of tumour inoculation and systemic injection is shown in Fig. 5A. All the mice survived well after cell implantation or were treated with vehicle, RSL3, cetuximab, or RSL3 in combination with cetuximab. Following treatment for 17 days, the size of the tumour gradually increased in the control and cetuximab treatment group. However, administration of RSL3 alone and treatment with both agents led to a decrease in tumour size, and the in the both agents’ administration group exhibited the greatest reduction in tumours size (Fig. 5B, C) and tumour volume (Fig. 5D). Body weight and daily food intake did not change significantly in the control or treatment groups (Fig. 5E). We then tested whether combinatory treatment with cetuximab and RSL3 can regulate lipid peroxidation in vivo. Strikingly, MDA concentration levels were significantly enhanced in the combined group (Fig. 5F).

**Fig. 6:**
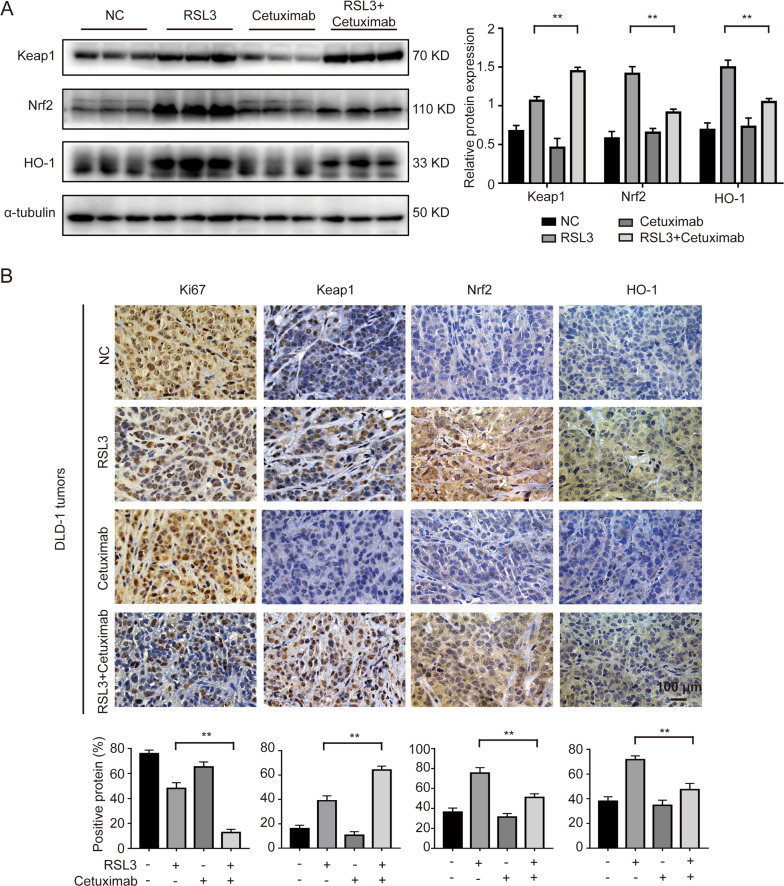
Cetuximab enhances RSL3-induced ferroptosis by inhibiting the Nrf2/HO-1 axis in vivo. **A** The protein levels of keap1, Nrf2 and HO-1 in tumour tissues were measured by western blotting. **B** The expression of Ki67, Keap1, Nrf2 and HO-1 were detected by immunohistochemical staining. Scale bar, 100 μm. **P < 0.01.

Next, we measured Nrf2, HO-1 and Keap1 protein levels. The western blotting date showed that the relative levels of Nrf2 and HO-1 were decreased and that keap1 expressed was relatively increased after co-treatment with RSL3 and cetuximab, as was observed in vitro (Fig. 6A). Then, we measured the ki67, Keap1, Nrf2, and HO-1 expression by immunohistochemical staining. Consistent with the western blotting results, the immunohistochemical analysis showed that low expression of Ki67, Nrf2, and HO-1 and high expression of keap1 were found after co-treatment with RSL3 and cetuximab (Fig. 6B). Taken together, these in vivo results further support the in vitro evidence that cetuximab enhances RSL3-induced ferroptosis by suppressing Nrf2/HO-1 activation.

## Discussion

CRC is the most common primary malignancy of the colorectum and progresses rapidly [23]. Moreover, KRAS is the most common form of mutated oncogene in cancer, and the frequency of KRAS mutations is particularly high in colorectal, pancreatic, and lung cancers [24–26]. Cetuximab, an EGFR-targeting agent, is a standard treatment for KRAS wild-type mCRC [27]. KRAS mutant CRC cells have been reported that it could promote tumour progression and induce resistance to cetuximab therapy [28]; however, there is no effective treatment to specifically treat cancers expressing KRAS mutations [29, 30].

Despite the failure of cetuximab in treating KRAS mutant CRC, development efforts and substantial research in this area have continued. Suto et al. confirmed that miR-7 regulates the sensitive of cetuximab by inhibiting EGFR signalling in KRAS mutant CRC cells [31]. Napolitano [32] and Troiani [33] et al. showed that co-treatment with cetuximab and regorafenib induced synergistic antiproliferative and apoptotic effects to overcome resistance to cetuximab by inhibiting MAPK and AKT pathways. Han et al. demonstrated that high expression of MLH1 increase cetuximab sensitivity by blocking HER-2 signalling [34].

Recently, research has shown that activation of ferroptosis effectively prevents tumour progression and enhances the efficacy of targeted therapy and chemotherapy. For example, it has been reported that vitamin C can lead to ferroptosis, and co-treatment with vitamin C and cetuximab could restrain the emergence of acquired resistance to cetuximab in RAS/BRAF wild-type CRC [35]. Upregulation of MT-1G expression protects hepatocellular carcinoma cells from the effect of sorafenib and promotes cancer progression by inhibiting ferroptosis [36]. Short-treatment of erastin significantly increases the cytotoxic effects of cisplatin [37]. The combination of a low concentration of paclitaxel and RSL3 induces ferroptosis in mutant p53 hypopharyngeal squamous cell carcinoma [38]. The combination of β-elemene, a bioactive compound, and cetuximab is effective to KRAS mutant CRC cells by inducing ferroptosis [39]. Additionally, in our study, we found that the cetuximab enhanced the cytotoxic effect of RSL3 on KRAS mutant CRC cells. Ferroptosis agent, but not the inhibitor of caspase, autophagy, or necroptosis, rescued the cell death induced by co-treatment with RSL3 and cetuximab, indicating that in the presence of cetuximab, ferroptosis is a key process that contributes to cell death. Interestingly, we found that cetuximab promoted RSL3-induced ferroptosis in KRAS mutant CRC cells.

Mechanistically, we demonstrated that Nrf2/HO-1 is essential for cetuximab to promote RSL3-induced ferroptosis in KRAS mutant CRC cells. Disruption of the Nrf2/HO-1 axis was previously shown to contribute to the promotion of ferroptosis [40–42]. In this study, we assessed the expression of Nrf2/HO-1 both in vitro and in vivo. The administration of cetuximab to RSL3-treated cells further promoted ferroptosis by decreasing Nrf2 and HO-1 expression. We determined that treatment with cetuximab significantly enhanced the cytotoxicity of RSL3 in KRAS-mutated CRC cells and promoted RSL3-induced ferroptosis by suppressing Nrf2/HO-1 expression. These results indicated that cetuximab decreased the activation of Nrf2/HO-1 in RSL3-treated cells, which could in turn increased RSL3-induced lipid ROS and MDA levels. In addition, the p38 MAPK pathway was evaluated in this study. p38 MAPK is the upstream molecule of Nrf2 [43, 44]. Previous studies have shown that nitric oxide induces HO-1 expression in HeLa cells [45] and that andrographolide induces Nrf2 and HO-1 expression in astrocytes [46], partly *via* p38 MAPK. In addition, p38 MAPK plays an important role in regulating the expression of Nrf2/HO-1 in brain astrocytes [47]. Ferroptosis has a broad clinical application prospect in cancer therapy [9]. In our study, we found that cetuximab combined with ferroptosis inducer treatment was effective in KRAS mutations CRC. Future preclinical studies are remine necessary to test whether ferroptosis-triggering agent combined with cetuximab could intensify the efficacy in KRAS mutant CRC.

**Fig. 7:**
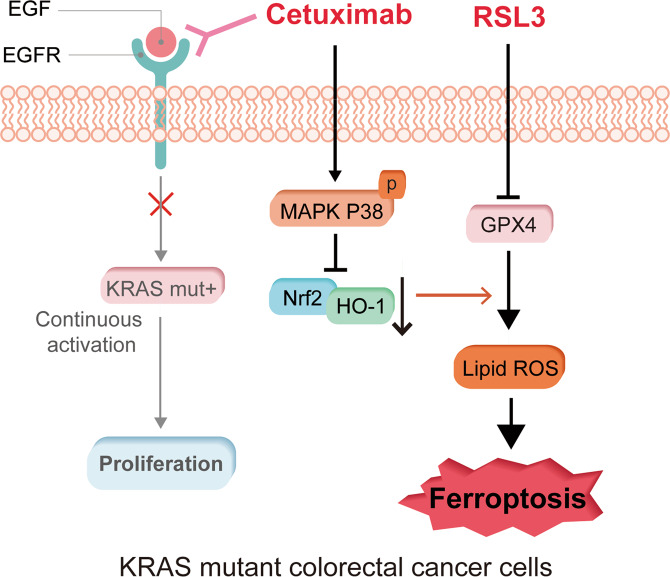
Graphical abstract of the mechanism by which cetuximab enhances RSL3-induced ferroptosis by inhibiting the Nrf2/HO-1 axis through the activation of p38 MAPK. Cetuximab inhibited the Nrf2/HO-1 pathway via p38 MAPK activation and then regulated the sensitivity to RSL3 in KRAS mutant CRC cells.

In conclusion, our study revealed that cetuximab enhances the cytotoxic effect of RSL3 on KRAS mutant CRC cells, and we demonstrated a novel mechanism involved in the anticancer effect of cetuximab on KRAS mutant CRC. Cetuximab enhanced RSL3-induced ferroptosis by inhibiting the Nrf2/HO-1 axis through the activation of p38 MAPK (Fig. 7). Our data suggests that it has the potential to be translated into clinical trials testing the effects of cetuximab combined treatment with ferroptosis inducer in KRAS mutant CRC patients. This finding will hopefully help in the development of an attractive treatment strategy for KRAS mutant CRC patients.

## Materials and methods

### Cell lines

Human colorectal cancer cell lines HCT116, DLD-1, LOVO and SW480 were obtained from the Chinese Academy of Sciences (Shanghai, China). HCT116 cells were cultured in DMED (Thermo Fisher Scientific, Waltham, MA, USA) supplemented with 10% foetal bovine serum (Thermo Fisher Scientific). DLD-1, LOVO and SW480 cells were cultured in RPMI 1640 medium (Thermo Fisher Scientific) supplemented with 10% FBS. The cells were maintained in a 5% CO_2_ humidified incubator at 37 °C.

### Reagents and antibodies

Cetuximab was purchased from Merck (Darmstadt, Germany), and RSL3 (HY-100218A) and SB202190 (HY-10295) were purchased from MedChemExpress (Monmouth Junction, NJ, USA). Necrostatin-1 (GC11008) and Z-VAD-FMK (GC12861) were purchased from GLPBIO (Montclair, CA, USA). Ferrostatin-1 (Fer-1) (HY-100579) and 3-MA (HY-19312) were purchased from MedChemExpress. Primary antibodies against Keap1 (AF5266), HO-1 (AF5393), p38 (BF8015), and phosphorylated (p)-P38 (AF4001) were purchased from Affinity Biosciences (OH, USA). A Nrf2 antibody (16396-1-AP) was purchased from Proteintech (Rosemont, IL, USA). A Ki67 antibody (ab15580) was purchased from Abcam (Cambridge, United Kingdom).

### Cell viability assay

The viability of CRC cells was determined by a Cell Counting Kit 8 (CCK-8, Dojindo, Japan). In brief, cells (3000-5000 per well) were plated in 96-well plates. Then, 100 μl of medium was added to cells containing 10 μl of CCK-8 solutions incubated at 37 °C for 3 h. Absorbance was measured at 450 nm wavelength.

### Western blot analysis

Protein concentrations were determined using a BCA Protein Assay Kit (Thermo Fisher Scientific). Proteins (30 μg) were electrophoresed on 12% SDS-PAGE gels and were transferred to PVDF membranes, which were blocked in 5% BSA for 2 h and were incubated with primary antibodies at 4 °C overnight. Then, carefully transferred to secondary antibody for 1 h. Next, we used TBST solution to wash the membranes three times. Antibody signals were detected using an ECL detection system (Bio-Rad, California, USA).

### Colony formation assays

To assess the colony formation of monolayer cultures, cells (1000 per well) were seeded into a 6-well plate. After two weeks, colonies were fixed and stained with 4% paraformaldehyde and 0.1% crystal violet for 30 min at room temperature.

### Lipid reactive oxygen species (ROS) level assay

Lipid ROS was detected by C11-BODIPY® 581/591 (D3861, Thermo Fisher Scientific) [48]. After 24 h, C11-BODIPY was incubated in cells with medium at 37 °C for 30 min. The the results were analyzed with FlowJo V10 software [39].

### Malondialdehyde (MDA) assay

MDA levels were measured with a Lipid Peroxidation MDA Assay Kit (#A003-4-1, Nanjing Jiancheng, China) according to the manufacturer’s instructions.

### Intracellular iron assay

Intracellular iron levels were assessed by using FerroOrange (Dojindo, Japan) [49]. Cells were seeded in 6-well plates and treated with the indicated treatment. After 24 h, FerroOrange (1 mM) dispersed in serum-free medium was added to the cells, and the cells were incubated for 30 min at 37 °C. Finally, the fluorescence was detected by confocal microscope.

### Small interfering RNA (siRNA) transfection

siRNA-Nrf2 or siRNA-Keap1 (GenePharma, Shanghai, China) were used to knocked down Nrf2 and Keap1. The sequences of the siRNAs: si-Nrf2-1 (sense: 5′-GCAUGCUACGUGAUG AAGATT-3′; antisense: 5′-UCUUCAUCACGUAGCAUG CTT-3′) and si-Nrf2-2 (sense: 5′-UCUUCAUCACGUAGCAUGCTT-3′; antisense: 5′-GCAUGCUAC GUGAUGAAGATT-3′), siKeap1-1 (sense: 5′-CCUCAAUCGUCUCCUUUAUTT-3′; antisense: 5′-AUAAAGGAGACGAUUGAGGTT-3′) and siKeap1-2 (sense: 5′-GUCCUGCA CAACUGUAUCUTT-3′; antisense: 5′-AGAUACAGUUGUGCAGGACTT-3′). Transfection was conducted with Lipofectamine 3000 (Thermo Fisher Scientific) according to the instructions of the manufacturer.

### Transduction

The vector expressing Nrf2 or HO-1 gene was obtained from GenePharma. HCT116 and DLD-1 cells were transient transfected with Nrf2 or HO-1 expressing vector. The upregulated expression of Nrf2 or HO-1 was confirmed by western blotting.

### Xenograft tumour models

The DLD-1 cell suspension (4 × 10^6^ cells/200 μl) was injected subcutaneously into the right dorsal flank of 5-week-old male BALB/c nude mice (Charles River, China). The mice were randomly divided into four groups (5 mice/group): 1) the control group, 2) the RSL3 group, 3) the cetuximab group, and 4) the RSL3 + cetuximab group. Both RSL3 (5 mg/kg) and cetuximab (13 mg/kg) were administered by intraperitoneal injection in a volume of 100 μl once per day. The tumour volume was calculated as 0.5 × length × width^2^ [50]. After 17 days of treatment, the mice were sacrificed, and the tumours were removed. Then, tumour tissue obtained from the different treated groups was subjected to western blotting and immunohistochemical experiments. The experiment was approved by the First Affiliated Hospital of Wenzhou Medical University Experimental Animal Ethics Committee.

### Immunohistochemistry (IHC)

Xenograft tumour tissues were fixed, dehydrated, embedded in paraffin, and sectioned (4 μm). The slides were incubated with the following primary antibodies: Ki67 (1:400), Keap1 (1:600), Nrf2 (1:50), and HO-1 (1:300).

### Statistical analysis

Statistical analysis was performed with GraphPad Prism 7.0 software (San Diego, CA, USA). *p* < 0.05 and *p* < 0.01 were defined as statistically significant and highly significant, respectively, compared with the control or indicated group. *p*-values were analyzed by using Student’s *t* test or one-way ANOVA. All data were generated from three independent experiments.

## Supplementary information

Supplemental data

Author Contribution Statement

## Data Availability

All data are included in this article and its supplementary materials or available upon request to the corresponding author.
