# Supplementary material for: Cetuximab promotes RSL3-induced ferroptosis by suppressing the Nrf2/HO-1 signalling pathway in KRAS mutant colorectal cancer
Source: Cell Death Dis. 2021 Nov 13;12(11):1079. doi: 10.1038/s41419-021-04367-3 (PMC8590697; doi:10.1038/s41419-021-04367-3)
Supplement: Supplementary file 1 — Supplemental data [file 41419_2021_4367_MOESM1_ESM.docx]

**Supplemental data**


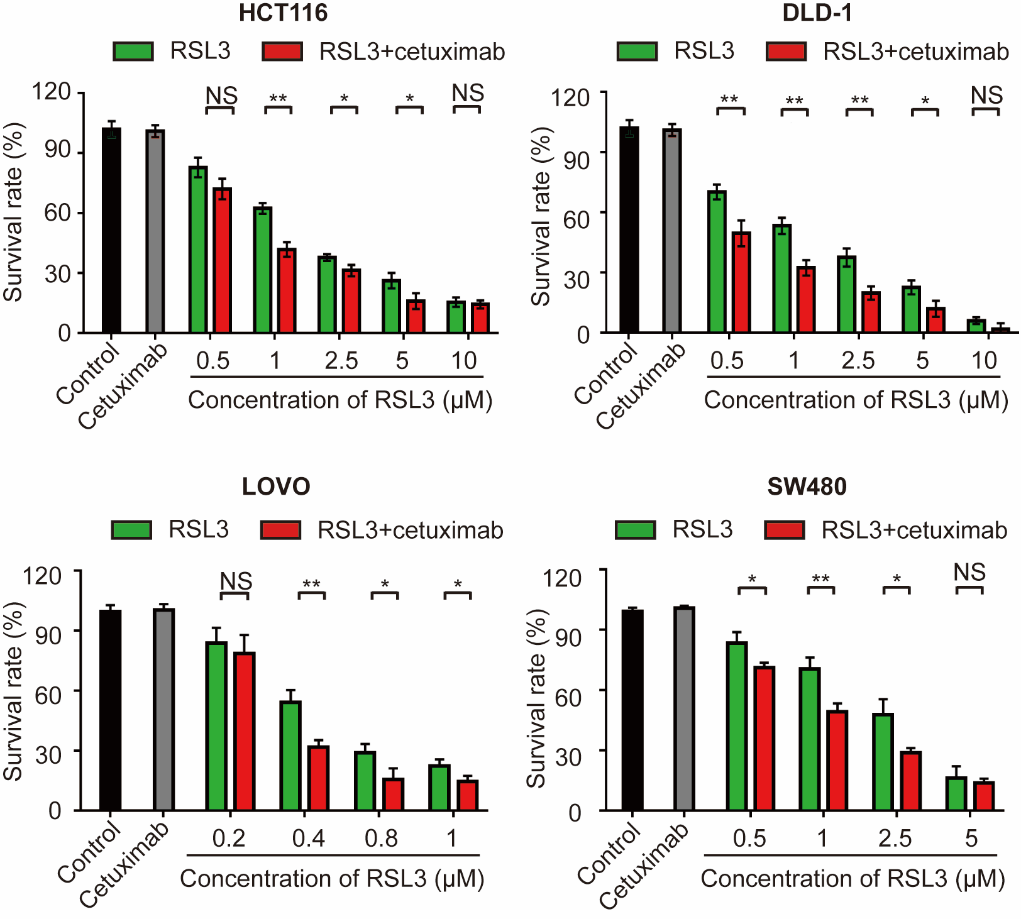


**Fig. S1 Effect of cetuximab combined with RSL3 in CRC cells.** Four KRAS mutant CRC cell lines (HCT116, DLD-1, LOVO and SW480) were treated with increasing concentrations of RSL3 alone or in combination with cetuximab (100 μg/ml) for 24 h. The inhibitory effects were determined by the CCK-8 assay. NS, not significant, **P* < 0.05, ***P* < 0.01.


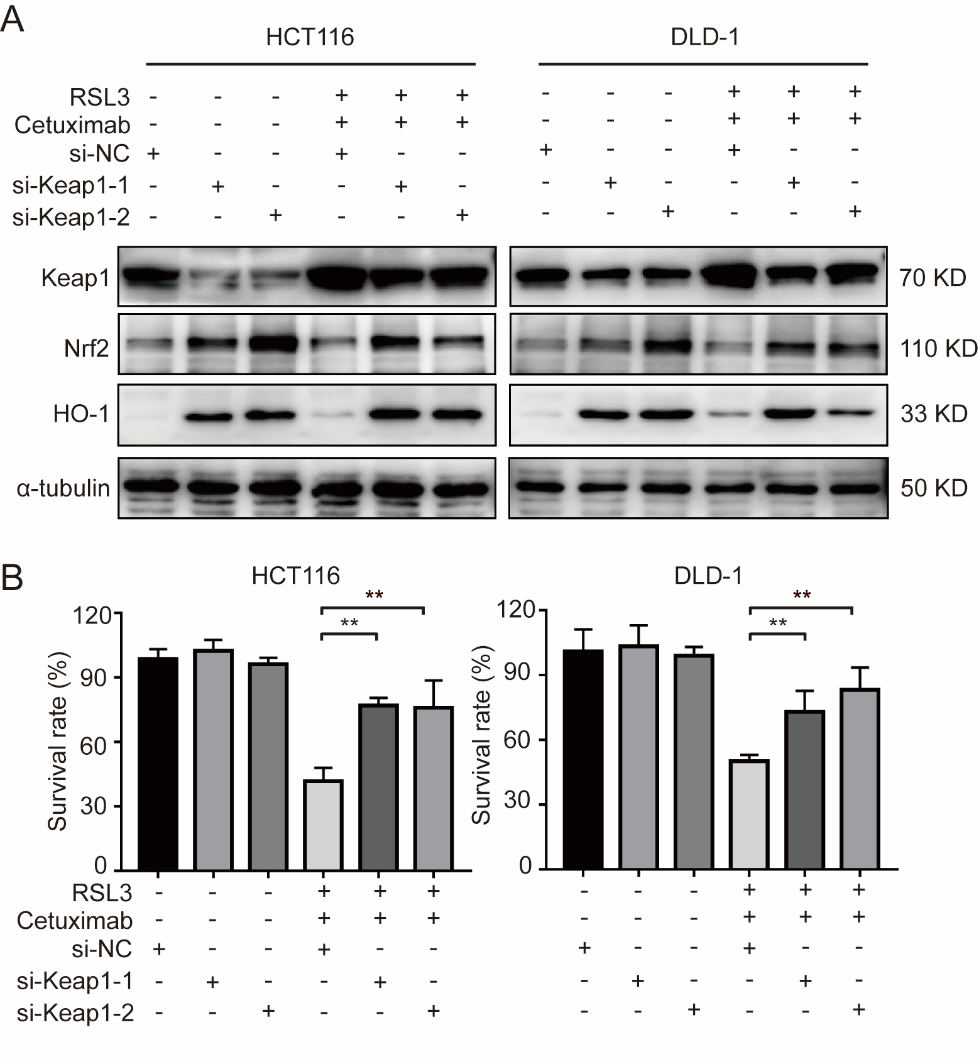


**Fig. S2 Knockdown of Keap1 restores the expression of Nrf2/HO-1 after combination treatment with RSL3 and cetuximab.** **A** Knockdown of Keap1 by siRNA restored the expression of Nrf2 and HO-1 after treatment with RSL3 (1 μM) and cetuximab (100 μg/ml) for 24 h. **B** knockdown of Keap1 partly reduced the cytotoxicity induced by combination treatment with cetuximab (100 μg/ml) and RSL3 (1 μM) in HCT116 and DLD-1 cells, and cell viability was assessed by the CCK-8 assay. ***P* < 0.01.
