# Supplementary material for: Cetuximab promotes RSL3-induced ferroptosis by suppressing the Nrf2/HO-1 signalling pathway in KRAS mutant colorectal cancer
Source: Cell Death Dis. 2021 Nov 13;12(11):1079. doi: 10.1038/s41419-021-04367-3 (PMC8590697; doi:10.1038/s41419-021-04367-3)
Supplement: Supplementary file 2 — Author Contribution Statement [file 41419_2021_4367_MOESM2_ESM.pdf]

DECLARATION OF CONTRIBUTIONS TO ARTICLE

ADMC

Manuscript Number: CDDIS-21-1979RR

Journal Name: *Cell Death & Disease* (the 'Journal')

Proposed Title of the Contribution:

Cetuximab promotes RSL3-induced ferroptosis by suppressing the Nrf2/HO-1 signaling pathway in KRAS mutant colorectal cancer (the 'Contribution)

Author(s):

Jiawen Yang, Jiajie Mo, Juji Dai, Chenqiao Ye, Wei Cen, Xuzhi Zheng, Lei Jiang and Lechi Ye (the 'Authors')

For all *CDDis* articles, each person named as an author in the published version must be able to show he or she has contributed substantially to the article.

Authorship credit should be based on 1) substantial contributions to conception and design, acquisition of data, or analysis and interpretation of data; 2) drafting the article or revising it critically for important intellectual content; and 3) final approval of the version to be published. Authors should meet conditions 1, 2 and 3.

Any person who cannot be shown to have made a substantial contribution to the article cannot be listed as an author in the final version. The name of any person who is deemed to have made a minor contribution can, however, appear in the Acknowledgments section of the article.

Please complete the table below to indicate the contributions of all named authors to the manuscript.

Author Full Name:	Specification of Contribution to the Manuscript:
Jiawen Yang	Jiawen Yang finished the experiments and draft the manuscript.
Jiajie Mo	Jiajie Mo revised the manuscript and participated in the experiments.
Juji Dai	Juji Dai participated in the experiments and data analysis.
Chenqiao Ye	Chenqiao Ye participated in the experiments and data analysis.
Wei Cen	Wei Cen participated in the experiments and data analysis.
Xuzhi Zheng	Xuzhi Zheng participated in the experiments and data analysis.
Lei Jiang	Lei Jiang designed experiments and modified the manuscript.
Lechi Ye	Lechi Ye designed experiments and modified the manuscript.

Please complete the table below to indicate the contributions of all named authors to the figures.

Figure 1:

Jiawen Yang finished the cell viability assay and colony assay.
Jiajie Mo taken representative cell morphological changes by inverted light microscopy.

Figure 2:

Jiawen Yang finished the lipid reactive oxygen species (ROS) accumulation, oxidative stress marker malondialdehyde (MDA) and intracellular iron assay

Figure 3:

Jiawen Yang finished the Western blot and cell viability assay and detected the lipid ROS, MDA levels.
Chenqiao Ye and Wei Cen were involved in knockdown of Nrf2 by siRNA and detecting the intracellular iron levels.

Figure 4:

Lei Jiang and Xuzhi Zheng assembled the figure.

Figure 5:

Jiawen Yang finished the animal experiments.
Jiajie Mo and Chenqiao Ye participated in data analysis.

Figure 6:

Lechi Ye and Juji Dai analyzed the data.

Signed for and on behalf of the Author(s):	Print Name:	Date:
Lechi Ye	Lechi Ye	2021.10.21
